# Epigenetics in Glaucoma

**DOI:** 10.3390/medicina60060905

**Published:** 2024-05-29

**Authors:** Fabiana D’Esposito, Caterina Gagliano, Philip Anthony Bloom, Maria Francesca Cordeiro, Alessandro Avitabile, Giuseppe Gagliano, Ciro Costagliola, Teresio Avitabile, Mutali Musa, Marco Zeppieri

**Affiliations:** 1 Imperial College Ophthalmic Research Group (ICORG) Unit, Imperial College, London NW1 5QH, UK; f.desposito@imperial.ac.uk (F.D.);; 2 Eye Clinic, Department of Neurosciences, Reproductive Sciences and Dentistry, University of Naples Federico II, 80131 Naples, Italy; 3 Department of Medicine and Surgery, University of Enna “Kore”, Piazza dell’Università, 94100 Enna, Italy; 4 Eye Clinic, Catania University San Marco Hospital, Viale Carlo Azeglio Ciampi, 95121 Catania, Italy; 5 Western Eye Hospital, Imperial College Healthcare NHS Trust, London NW1 5QH, UK; 6 Department of Optometry, University of Benin, Benin City 300238, Nigeria; 7 Department of Ophthalmology, University Hospital of Udine, 33100 Udine, Italy

**Keywords:** open angle glaucoma, epigenetics, DNA methylation, histone modifications, non-coding RNAs, glaucomatous neurodegeneration

## Abstract

Primary open angle glaucoma (POAG) is defined as a “genetically complex trait”, where modifying factors act on a genetic predisposing background. For the majority of glaucomatous conditions, DNA variants are not sufficient to explain pathogenesis. Some genes are clearly underlying the more “Mendelian” forms, while a growing number of related polymorphisms in other genes have been identified in recent years. Environmental, dietary, or biological factors are known to influence the development of the condition, but interactions between these factors and the genetic background are poorly understood. Several studies conducted in recent years have led to evidence that epigenetics, that is, changes in the pattern of gene expression without any changes in the DNA sequence, appear to be the missing link. Different epigenetic mechanisms have been proven to lead to glaucomatous changes in the eye, principally DNA methylation, post-translational histone modification, and RNA-associated gene regulation by non-coding RNAs. The aim of this work is to define the principal epigenetic actors in glaucoma pathogenesis. The identification of such mechanisms could potentially lead to new perspectives on therapeutic strategies.

## 1. Introduction

The term “glaucoma” refers to a wide spectrum of different eye disorders which ultimately lead to irreversible damage to retinal ganglion cells (RGCs) with progressive optic neuropathy [1,2]. The most common form is primary open angle glaucoma (POAG), affecting approximately 3.5 percent of the world population aged 40 to 80 years, and most prevalent in individuals of African descent [3,4,5].

Most forms of glaucoma are associated with an increase in intraocular pressure (IOP), causing mechanical damage within the eye, ischaemia, oxidative stress, and damage to the optic nerve [4]. At present, most therapeutic strategies aim to lower IOP, either pharmacologically or by surgical or para-surgical (laser) interventions [6]. New drugs are being developed in the field of neuroprotection [7,8,9], but the real challenge is to identify the complex mechanisms leading to the condition in order to identify potential therapeutic targets and establish focussed therapies.

In different forms of glaucoma, genetics play a specific role [10] to variable degrees. In particular, the forms that are early-onset or related to developmental disorders of the anterior segment can be caused by frankly pathogenic DNA variants inherited according to “Mendelian” patterns in a variety of genes such as *MYOC*, *CYP1B1*, *PAX6*, *FOXC1*, and *PITX2* [11] (Table 1).

Genetic studies on families with significant numbers of affected individuals have led to the identification of at least 15 genes or chromosomal loci (GLC1A-P) [11] involved in the pathogenesis. Among those, a large number of genes play a clear determining role in the development of the disease, including myocilin (*MYOC*), optineurin (*OPTN*), WD repeat domain 36 (*WDR36*), ankyrin repeat and SOCS-box containing 10 (*ASB10*), cytochrome P450 family 1, subtype B, polypeptide 1 (*CYP1B1*), EGF-containing fibulin-like extracellular matrix protein 1 (*EFEMP1*), and neurotrophin 4 (*NTF4*). *MYOC* variants are thought to be the underlying cause of 3–4% of POAG cases with IOP > 21 mmHg, while pathogenic variants in *OPTN*, *OPA1*, and *MYOC* each appear to cause ∼1% of normal tension glaucoma (NTG) cases [12].

Despite the numerous genes and loci identified to underlie glaucoma, POAG, in particular, is considered a “genetically complex trait”, where genes play an important but not unique role in its manifestation [13].

The DNA sequence has a degree of variability among individuals, giving rise to unique characteristics. Variants in the DNA can be neutral, frankly pathogenic (giving rise to the “Mendelian” traits), or polymorphic, the so-called single-nucleotide polymorphisms (SNPs). Polymorphisms are single-nucleotide DNA variations that have potentially different roles in the development of pathologic conditions: neutral, predisposing, or protective [14].

In addition to the above-mentioned genes with a mendelian form of inheritance, Genome-wide association studies (GWASs) have led to the identification of a consistent number of polymorphisms in genes implicated in the pathogenesis of glaucoma [15].

New concepts of genetic risk scores (GRSs) and polygenic risk scores (PRSs) are now also being applied to glaucoma pathogenesis [16]. GRS refers to the presence of variants with a statistically significant association at the genome-wide level. PRS combines the presence of different variants to define a single individual’s risk of manifesting a disease [17,18,19,20].

Although much progress has been made in elucidating genetic mechanisms underlying glaucoma, a clearly genetic cause can only be defined in a small percentage of patients, leading to the conclusion that different mechanisms play a role together with genetic status. This conclusion is supported by various studies that highlight the importance of additional factors beyond purely genetic determinants [13].

Another aspect suggesting that classical genetic mechanisms are not the only causative factors in the pathogenesis of glaucoma is the observation that the prevalence of this condition is steadily increasing over the years independently of the availability of better diagnostic tools.

This rising prevalence indicates that several lifestyle and environmental factors play a significant role. The main defined factors include ageing, inflammation, oxidative stress, drugs, and diet [21]. The manner in which these factors interact with an individual’s DNA and lead to altered gene expression can be explained by various epigenetic mechanisms.

The term epigenetics refers to mitotically heritable changes in the pattern of gene expression without any changes in the DNA sequence, while the term epigenomics denotes the study of epigenetics on a genome-wide basis [22,23,24]. These definitions underscore the importance of understanding how gene expression may be regulated by factors other than direct genetic variants.

Epigenetic effects are now recognised as having a well-established role in ophthalmology, playing a critical part through the regulation of gene expression during normal eye development and the regulation of the metabolism of eye structures throughout an individual’s lifetime [25]. This regulatory function is essential for maintaining the health and normal function of ocular tissues. Recent studies have led to the identification of several epigenetic mechanisms that are involved in the development of some of the most significant ocular diseases such as cataract, glaucoma, ocular surface disorders [21], and age-related macular degeneration [26]. These discoveries have provided valuable insights into how these diseases develop and progress at the molecular level.

In the context of glaucoma, one of the most significant and prevalent eye conditions, several key epigenetic mechanisms have been identified. These include DNA methylation, which involves the addition of methyl groups to the DNA molecule, thereby affecting gene expression without altering the underlying DNA sequence. This process is crucial for regulating genes involved in eye health and disease [4,27]. Post-translational histone modification is another significant mechanism, involving the chemical modification of histone proteins around which DNA is wound. These modifications can change how tightly or loosely DNA is packaged, thus influencing gene expression.

Chromatin remodelling is also a vital epigenetic mechanism in glaucoma, referring to the dynamic modification of the chromatin structure to allow access to genetic material, thereby enabling the activation or repression of genes as needed. Additionally, RNA-associated gene regulation by non-coding RNAs, including microRNAs and long non-coding RNAs, plays a critical role. These RNA molecules can regulate gene expression at transcriptional and post-transcriptional levels, contributing to the complex regulatory networks that maintain ocular health and mediate disease processes [4,27].

Together, these epigenetic mechanisms not only enhance our understanding of the molecular basis of glaucoma but also highlight potential targets for therapeutic interventions. By targeting specific epigenetic modifications it may be possible to develop novel treatments aimed at preventing or slowing the progression of glaucoma, thereby preserving vision and improving the quality of life for patients.

## 2. Epigenetic Modifications in Glaucoma

**Figure 1:**
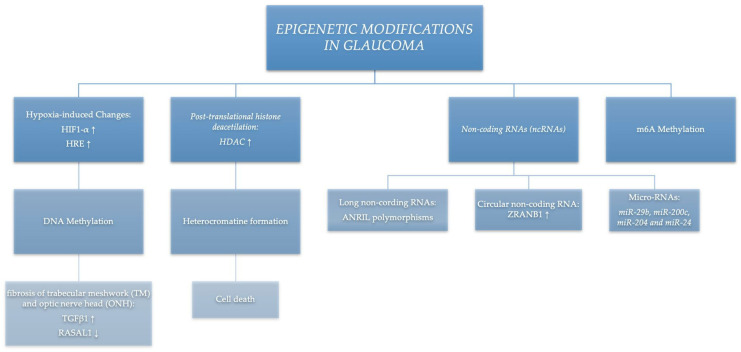
Epigenetic modifications in glaucoma.

A growing number of epigenetic mechanisms have been described in the literature as influencing the development of glaucoma. In this review we discuss the most relevant of these factors (summarised in Figure 1), bearing in mind that this is a rapidly expanding field with continual elucidation of new pathways.

### 2.1. Hypoxia-Induced Changes—DNA Methylation

Expression of different genes such as those involved in apoptosis, neurogenesis, extracellular matrix production, mitochondrial function, and angiogenesis can vary significantly under the effect of hypoxia levels [28]. This variation is crucial because low oxygen levels can influence a wide range of cellular processes and gene expression pathways, leading to various physiological and pathological outcomes. The glaucomatous eye is typically a hypoxic environment, primarily due to the defective outflow of aqueous humour [29], resulting in increased intraocular pressure which may compromise ocular blood flow and lead to hypoxia.

The hypoxic conditions within the glaucomatous eye exacerbate disease progression by affecting multiple cellular functions. Hypoxia inducible factor 1-α (HIF1-α) is a subunit of heterodimeric transcription factor hypoxia inducible factor 1 (HIF1) considered to have a pivotal role in the regulation of the response of cells to hypoxia, where HIF-1α expression and HIF1 transcriptional activity increase as cellular oxygen concentration decreases [30]. Tezel et al. [29] demonstrated that HIF1-α is overexpressed in the glaucomatous retina and optic nerve head, postulating a pathophysiologic role in the development and/or progression of neurodegeneration.

Hypoxia is also known to stimulate DNA methylation of the HIF response element (HRE) that enhances its binding to the HIF1-α and ultimately stimulates the transition of the cells in the trabecular meshwork from epithelial to mesenchymal [31], thereby leading to fibrosis [32].

DNA methylation is an epigenetic mechanism known to be involved in gene expression regulation and having an important role in several cellular processes such as embryonic development, X-chromosome inactivation, and preservation of chromosome stability. It occurs at the cytosine bases of eukaryotic DNA, which are converted to 5-methylcytosine by DNA methyltransferase (DNMT) enzymes. Abnormalities in methylation processes have been linked to several human diseases, including glaucoma [4].

The fact that methylation determines fibroblast activation and fibrogenesis has been well described by Bechtel et al. [33]. Extracellular matrix (ECM) deposition inducing fibrosis at the level of trabecular meshwork (TM) and optic nerve head (ONH) is known to be a pathological mechanism involved in the ultimate damage of RGCs and optic nerve fibres in glaucoma. Increased levels of TGFβ are known to be related to the abnormal production of ECM.

McDonnell et al. [34] studied the interactions between hypoxia-induced DNA methylation and the expression of fibrosis-related genes such as the pro-fibrotic transforming growth factor TGFβ1 and the anti-fibrotic Ras protein activator like1 (RASAL1). They compared the expression levels in human normal TM (NTM) cells with glaucomatous TM cells and with NTM cells under hypoxic conditions. Hypoxia alone was shown to alter the DNA methylation status of cells and influence levels of TGFβ1 and RASAL1 in the cells of both normal and glaucomatous trabecular meshwork. RASAL1 was shown to be downregulated directly by TGFβ1 and then indirectly by its promoter methylation through DNA methyltransferase1 enzyme (DNMT1) activity.

In conclusion, in the glaucomatous eye, hypoxia could be the trigger for increased levels of DNA methylation in related loci, consequently affecting the regulation of the expression of TGFβ1 (that stimulates fibrosis) and RASAL1 (a fibrosis inhibitor) [34].

The precise elucidation of a role for methylation and TGFβ1 regulation in the pathogenesis of glaucoma may provide valuable steering in the development of new potential therapeutic strategies that would go far beyond the current approach of merely lowering IOP.

### 2.2. Post-Translational Histone Modifications

Histones are proteins needed to package DNA in eukaryotic cell nuclei. They are subject to post-translational modifications, such as methylation, acetylation, or phosphorylation, that alter their interaction with DNA and nuclear proteins and play a role in gene expression [35].

Among the different mechanisms, DNA expression appears to be regulated by histone acetylation and deacetylation under the action of the specific enzymes histone acetyltransferases (HATs) and histone deacetylases (HDACs) [27].

Deacetylation is related to a more compact chromatin structure, thereby limiting the access of transcription factors to the DNA. Conversely, the addition of acetyl groups has the opposite effect, facilitating the interaction of chromatin with transcription factors by creating a more relaxed chromatin structure [36,37,38,39]. This dynamic modification of chromatin accessibility plays a crucial role in the regulation of gene expression.

Silencing of normal gene expression is considered to be an early event in cells undergoing apoptosis, including retinal ganglion cells (RGCs) [40]. Apoptosis, or programmed cell death, is a significant process in the pathology of glaucoma, leading to the loss of RGCs. Studies on modifications of the RGC axons after acute injury to the optic nerve have unveiled a series of mechanisms that can provide a better understanding of nerve degeneration occurring in glaucomatous eyes and the related epigenetic mechanisms. One of the critical pathways leading to nucleus atrophy and cell death involves histone deacetylation and subsequent heterochromatin formation, an essential step in this degenerative process.

In normal RGCs, histone deacetylases (HDACs) play a significant role. HDAC1 and HDAC2 are localised to the nuclei, where they are involved in maintaining chromatin structure and regulating gene expression. In contrast, HDAC3 is primarily localised to the cytoplasm. The distinct localisation of these HDACs suggests specific roles in the cellular processes of RGCs. Understanding these mechanisms provides valuable insights into potential therapeutic targets for preventing RGC death and preserving vision in glaucoma patients.

A murine model of optic nerve crush has been used to investigate variations of HDAC activity in the process of cell death. After acute optic nerve injury, mRNA accumulation of class I HDACs (and especially HDAC2 and HDAC3 expression) increases and HDAC3 translocates from the cytoplasm to the nucleus; this has also been demonstrated in other neurological diseases. At the same time histone H4 acetylase appears to be upregulated [27,36,37,40].

The elucidations of such mechanisms could be crucial. The experimental inhibition of retinal HDAC activity with specific agents such as valproic acid (VPA) has been proven to be successful as neuroprotective treatment [41,42,43,44,45].

Specifically, VPA is an inhibitor of the activity of HDAC enzymes, particularly HDAC1, and is involved in the regulation of gene expression by modifying the acetylation status of histones [44,45].

### 2.3. RNA-Associated Gene Regulation by Non-Coding RNAs (ncRNAs)

Non-coding RNAs are different classes of RNA molecules, such as long non-coding RNAs (lncRNAs), circular RNAs (circRNAs), and microRNAs (miRNAs), that do not encode functional proteins. It is estimated that they constitute about 60% of the genetic material in the human genome [46]. It is now known that they are involved in regulatory mechanisms of gene expression, potentially related to key regulators of several biological processes, and are implicated in the onset of different diseases. Their classification is complex, and a growing number are being characterised in terms of structure and function.

Non-coding RNAs have also been demonstrated to play a role in the pathogenesis of glaucoma [47,48]. Recent research has shed light on the mechanisms by which these non-coding RNAs influence gene expression and contribute to disease processes. For instance, miRNAs have been shown to regulate the expression of genes involved in intraocular pressure and optic nerve health, which are critical factors in glaucoma development. Similarly, lncRNAs and circRNAs have been implicated in cellular processes such as apoptosis and inflammation, further linking them to glaucoma pathogenesis.

Understanding the specific roles of these non-coding RNAs in glaucoma can provide new insights into potential therapeutic targets. By manipulating the expression or function of these RNAs, it may be possible to develop novel treatments that mitigate the progression of glaucoma and neurodegeneration. **Long non-coding RNAs (lncRNAs)** are non-protein-coding transcripts with a size ranging from 200 to 100,000 nucleotides. They have been described to have a role in the regulation of gene expression at various levels and are reportedly implicated in different disease aetiologies [49]. Their regulatory effects can be found at different levels, pre-transcriptional (i.e., binding to DNMT or regulating histone modifications), transcriptional (i.e., interacting directly with transcription factors), or post-transcriptional, targeting specific mRNAs [50].

One of the more thoroughly studied lncRNAs is the cyclin-dependent kinase inhibitor 2B antisense non-coding RNA (CDKN2B-AS1), also known as ANRIL. The identification of polymorphisms in ANRIL and subsequent genotype/phenotype studies revealed the existence of SNPs that are significantly related to entities of optic nerve neurodegeneration in POAG patients. From this result, it has been suggested that different isoforms of ANRIL can modify the vulnerability of the optic nerve and therefore modulate neurodegeneration. The risk alleles in the ANRIL region can predispose to the development of POAG at lower IOP levels as occurs in normal tension glaucoma [51,52].

Another interesting class of ncRNAs is the **circular RNAs (circRNAs)** that have been proven to act as potential regulators in different neurodegenerative disorders, probably through mechanisms of post-transcriptional gene regulation [53].

The circRNA ZRANB1 is mainly expressed in the cytoplasm of glial cells, indicating that its regulatory activity is at a post-transcriptional level. It has been demonstrated by Wang et al. [48] that its expression is significantly upregulated in retinal degeneration induced by glaucoma. It appears to have a negative control effect on the expression of miR-217 with the effect of an increase in Müller cell proliferation. Interestingly, they studied the effects of cZRANB1 knock-down on in vivo glaucoma-induced eyes, resulting in both the decrease in retinal gliosis and apoptosis rate in the RGCs. Furthermore, they demonstrated that the overexpression of transcription factor RUNx2 can reverse the effects of cZRANB1 knock-down, identifying in the intervention on the cZRANB1/miR-217/RUNX2 signalling network a potential effective therapeutic strategy to treat glaucoma-related retinal neurodegeneration [53].

**MicroRNAs (miRNAs)**. The trabecular meshwork (TM) is the structure that drains aqueous humour (AH) from the anterior chamber, thereby modulating an optimal intraocular pressure.

miRNAs may have a role in tissue remodelling [54], therefore influencing the resistance to flow in the TM by altering its constitution and particularly by inducing the differentiation of myoblasts into myofibroblasts in glaucomatous eyes [55,56,57,58]. Additionally, miRNAs such as miR-149e, miR-93-5p, miR-141-3, and miR211 may intervene in the regulation of retinal ganglion cell (RGC) metabolism [48]

Several studies have focussed on differential levels of miRNA expression in the comparison between the aqueous humour of normal and glaucomatous eyes [48,59]. Particularly, miR-29b, miR-200c, miR-204, and miR-24 have been characterised as potential biomarkers for glaucoma diagnosis and characterisation [60]. The identification of miRNA-related mechanisms involved in the pathogenesis of glaucoma represents a particularly valuable tool, characterising a specific target in the potential development of future therapies.

### 2.4. M6A Methylation

N6-methyladenosine (m6A) methylation is the most prevalent post-transcriptional modification in eukaryotic mRNA and long non-coding RNA, comprising the addition of a methyl group at the N6 position of adenosine [61,62].

M6A RNA methylation is a dynamic and reversible process that modulates physiological processes such as gene expression, maintenance of homeostasis, and stem cell differentiation. In addition, a regulatory effect has been described in pathological processes such as inflammation, angiogenesis, degeneration, and more. M6A methylation is controlled by different factors, defined as “writers” (METTL3, METTL14, METTL16, WTAP, KIAA1429, RBM15, and ZFP217), “erasers” (FTO and ALKBH5), and “readers” (YTHDF1,2,3, YTHDC1,2, eIF3, IGF2BP1,2,3, HNRNPA2B1, FMR1, and LRPPRC) [63].

The regulation of m6A factors has been extensively studied in traumatic optic nerve injury (a model for glaucoma) in its RGC degeneration process [46].

**Key Points:**

- Glaucoma is considered a “genetically complex trait”, with a growing number of identified related genes.
- Genetic variations alone only can only explain a limited fraction of cases.
- External factors can interact with the genetic background through epigenetic mechanisms.
- The major better-defined epigenetic mechanisms related to glaucoma act through: DNA methylation, histone modifications, and non-coding RNAs.
- Identification of epigenetic mechanisms related to glaucoma may lead to alternative therapeutic approaches.

## 3. Conclusions

The term “glaucoma” comprises several different pathological entities, resulting in a final common pathway of damage to retinal ganglion cells and optic nerve fibres. Amongst those, the pathogenetic impact of the genetic background varies by subtype of glaucoma [64].

In open angle chronic glaucoma, it is well known that there is a genetic predisposition by family and/or by race, indicating a strong genetic component; this can only be precisely defined in a very limited proportion of patients carrying pathogenic variants of specific genes. At the same time, it has been found that heavy smoking, exposure to pesticides, nutrient intake, intrauterine environment (obesity and diabetes), and other factors can affect later development of glaucoma.

As for exfoliation-syndrome-related or pseudo-exfoliative glaucoma, geographic and climatic factors such as sun exposure and ambient temperature are considered to play an important role, in addition to a possible genetic susceptibility [65].

**Figure 2:**
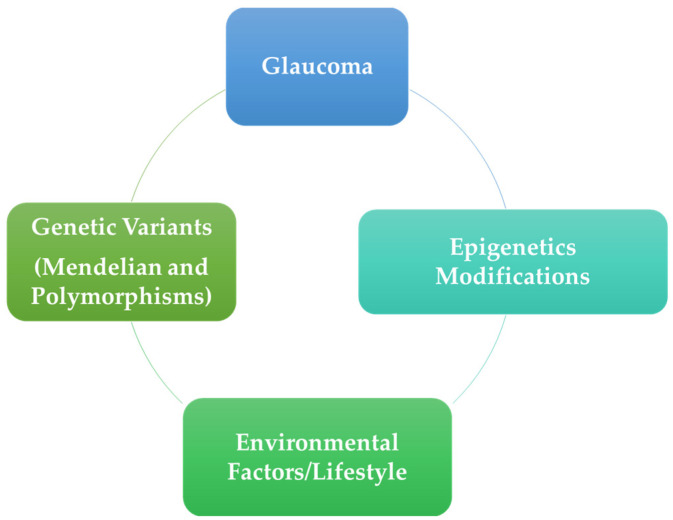
Interaction of factors in glaucoma pathogenesis.

In this complex scenario regarding the pathogenesis, we now know that yet more factors must be considered to explain the different roles of genetic and non-genetic factors and in particular the interactions between them. In recent years several epigenetic mechanisms have been elucidated in different human pathologic traits, demonstrating connections between genetics and environmental and/or lifestyle factors [46,66] (Figure 2).

Three broad strands of research continue to provide us with new results in the process of elucidating mechanisms leading to glaucoma: identification of variants in genes powerful enough to have a clearly pathogenic effect [67], GWASs aiming at the characterisation of predisposing or protective SNPs [68,69], and studies on the identification of epigenetic mechanisms [46,47].

Different influence of epigenetic factors, in particular, may go a long way to explain the high degree of variability observable among affected patients in different aspects of the disease such as susceptibility of the optic nerve fibres to IOP-related damage [70]. The extremes of variability are represented by normal tension glaucoma (NTG), where damage occurs despite low IOP, and ocular hypertension (OHT), where patients have high tolerance to raised IOP levels with no damage to the optic nerve.

Similarly, there is high individual variability in the IOP response to ocular application of topical steroids that can lead to IOP increase and steroid-induced glaucoma. This may be determined by individual characteristics related to the glucocorticoid receptor gene and/or to additional epigenetic mechanisms that enhance the biochemical cascade that leads to a rise in IOP [71,72].

Furthermore, while the pharmacology of glaucoma medications is well understood, it is not clear why individual patients respond differently to IOP-lowering drugs [73]. Several variables, including environmental factors (such as chemicals, alcohol, tobacco, diet, and other drugs) or biological factors (such as age and gender), may contribute to the physiological and biochemical status of the targeted cells and finally modify the expression of related genes. Again, the determinants of variable effects of glaucoma drugs, and even the phenomenon of tachyphylaxis (the reduced effectiveness of a medication over time), might be explained by epigenetic mechanisms activated by such factors. Therefore, it is not simply genetics or environment, but most likely the interplay between those factors, that is important in the development of glaucoma, its natural course, and the individual response to pharmacological treatments [46].

Recent years have seen a rapid expansion of knowledge in the field of epigenetics. Epigenetic mechanisms have been found to underlie both physiological and pathological conditions and may be the ultimate explanation for the pathogenesis of complex traits in conditions such as glaucoma.

A further important point driving much of the enthusiasm for studies aiming at the characterisation of epigenetic mechanisms is the possibility that epigenetic modifications could in principle be reversible; this raises the prospect of a completely new therapeutic approach, with the possibility of individualised medical treatments, as already suggested by some experimental data.

## Figures and Tables

**Table 1:** Principal glaucoma-related genes.

Gene	Glaucoma Subtype	Transmission	Protein
*MYOC*	POAG, NTG	AD	Myocilin
*OPTN*	NTG, POAG	AD	Optineurin
*WDR36*	POAG		WD repeat domain 36
*ASB10*	POAG	AD	Ankyrin repeat and SOCS-box containing 10
*CYP1B1*	POAG, CONG G, JUV G, ASD	AR	Cytochrome P450 family 1, subtype B, polypeptide 1
*EFEMP1*	POAG	AD	EGF-containing fibulin-like extracellular matrix protein 1
*NTF4*	POAG		Neurotrophin 4
*OPA1*	POAG		Mitochondrial dynamin-like GTPase

POAG: Primary Open Angle Glaucoma; NTG: Normal Tension Glaucoma; CONG G: Congenital Glaucoma; JUV G: Juvenile Glaucoma: ASD: Anterior Segment Dysgenesis; AD: Autosomal Dominant; AR Autosomal Recessive.

## Data Availability

Not applicable.
